# Children and Adolescents with Mucopolysaccharidosis and Osteogenesis Imperfecta: The Dentistry on the Multiprofessional Team

**DOI:** 10.3390/jpm15070323

**Published:** 2025-07-18

**Authors:** Mariana Laís Silva Celestino, Natália Cristina Ruy Carneiro, Heloisa Vieira Prado, Glória Maria Pimenta Cabral, Mauro Henrique Nogueira Guimarães Abreu, Ana Cristina Borges-Oliveira

**Affiliations:** 1 School of Dentistry, Department of Social and Preventive Dentistry, Universidade Federal de Minas Gerais, Avenida Antônio Carlos, 6627, Belo Horizonte 31270-901, MG, Brazil; mlscelestino@hotmail.com (M.L.S.C.); maurohenriqueabreu@gmail.com (M.H.N.G.A.); 2 Faculty of Dentistry, Anhanguera, Rua dos Timbiras, 1375, Funcionários, Belo Horizonte 30140-060, MG, Brazil; nataliacrcarneiro@hotmail.com; 3 Faculty of Dentistry São Leopoldo Mandic, Special Care Dentistry Residency, R. Dr. José Rocha Junqueira, 13, Pte. Preta, Campinas 13045-755, SP, Brazil; heloisaprado92@hotmail.com; 4 School of Dental Medicine, Department of Pediatric Dentistry, Boston University, 635 Albany St., Boston, MA 02118, USA; gloriapimenta2@hotmail.com

**Keywords:** rare diseases, dental health, patient care team, disabled children, dental care for disabled, Mucopolysaccharidoses, Osteogenesis Imperfecta

## Abstract

**Background/Objectives:** To identify factors associated with the referral by a multiprofessional team to dental services for children and adolescents with rare genetic diseases. **Methods:** A cross-sectional study was developed with 87 children/adolescents with mucopolysaccharidosis (*n* = 26) and osteogenesis imperfecta (*n* = 61) and their caregivers. Recruitment took place at reference centers for rare genetic conditions in five Brazilian states. The caregivers answered a questionnaire on the children. They were examined for malocclusion, dental anomalies, caries experience, and gingivitis. Bivariate and multivariate analyses of the data were performed, considering a 95% confidence level. **Results:** The average age of children/adolescents was 10.4 years (±5.6) and 17.3% had never gone to a dentist. Among those with past dental experience, the reason for most appointments was oral prophylaxis/preventive maintenance (62.1%). With regard to referrals to a dentist by the multidisciplinary team, 29.9% had never received a referral. The likelihood of having been referred to a dentist by the multiprofessional team was 2.67 times greater for female patients (95% CI: 0.96–7.42) and 7.74 times greater for children/adolescents with a history of toothache (95% CI: 1.61–37.14). **Conclusions**: Female children/adolescents with mucopolysaccharidosis and osteogenesis imperfecta and those with a history of dental pain were more likely to have been advised by the multiprofessional team to seek dental treatment.

## 1. Introduction

Rare diseases are chronic, degenerative, progressive, and even debilitating conditions [1]. A disease is considered rare when it affects 80 or fewer individuals in every 100 thousand [2,3]. However, such diseases are estimated to affect 10% of the population, with considerable epidemiological impact, making these conditions a significant public health problem [2,4]. In Brazil, an estimated 13 million individuals have some rare disease, and a substantial portion of people remain undiagnosed [5,6]. Approximately 71.9% of rare diseases are genetic and affect children, some with exclusively pediatric beginning. These conditions are often accompanied by physical, intellectual, emotional, and/or behavioral limitations [2,3,7].

Mucopolysaccharidosis (MPS) and Osteogenesis Imperfecta (OI) are rare diseases that affect skeletal development. The first one is a progressive condition that leads to the accumulation of glycosaminoglycans inside lysosomes of cells. The second one is a rare disease caused by mutations in COLIA1 and COLIA2 genes and leads to altered synthesis of collagen type 1 and consequently bone deformities. Due to the consequences of the conditions, these two populations of rare diseases are often faced with oral alterations such as facial growth alterations, dental anomalies, and malocclusion. Furthermore, such individuals often have difficulty in gaining access to adequate dental treatment, even in the private sector [4,8,9].

Considering the progressive characteristics of these two conditions and the oral and systemic problems faced by these patients, the health care requires a multidimensional view and should be provided by a team that includes health professionals from different fields, including the dentistry team [2,8,10,11]. Multiprofessional and interdisciplinary teams can perform collaborative activities that enable sharing information, coordinating care, identifying problems, developing intervention plans, and defining shared goals [12,13]. On top of that, the recognition of the oral problems faced by those patients is essential to provide a better quality of life for them. Thus, it is important that the health care providers be aware of that and advise guardians to seek dental treatment for their children as soon as possible.

The vulnerability of individuals with MPS and OI to the development of oral problems underscores the importance of early preventive dental assistance and the collaborative action of a multidisciplinary team. Therefore, the aim of the present study was to identify factors associated with the referral by the multiprofessional team to dental services for children and adolescents with two rare genetic diseases (MPS and OI).

## 2. Materials and Methods

### 2.1. Ethical Aspects

The present study received approval from the Human Research Ethics Committee of the Federal University of Minas Gerais, under the protocol numbers 01480212.4.0000.5149 [MPS], approved on 25 April 2013 and 54755516.4.0000.5149 [OI], approved on 31 August 2016.

### 2.2. Study Design and Sample

A cross-sectional design was employed and a convenience sample of children/adolescents with rare genetic diseases and their parents/guardians were recruited. Individuals between 2 and 21 years of age with a diagnosis of osteogenesis imperfecta (OI) or mucopolysaccharidosis (MPS) were included. OI and MPS are rare conditions that affect the formation and function of bones and joints and involve various orofacial abnormalities and oral problems [5,10,14]. To broaden the sample size, participants were recruited using the snowball sampling strategy [15]. The study was carried out between January and December 2019.

The sample was recruited from specialized care clinics for patients with rare genetic disorders in five Brazilian states (Ceará, Espírito Santo, Minas Gerais, Rio de Janeiro, and São Paulo). The locations were selected by geographic convenience and the feasibility of data collection. None of the locations offer dental care.

### 2.3. Inclusion and Exclusion Criteria

Participants were included based on the following inclusion criteria:

1. Individuals diagnosed with MPS, along with their parents or legal guardians.
2. Individuals diagnosed with OI, along with their parents or legal guardians.
3. Individuals with MPS or OI aged 2 years to 21 years and their respective parents or guardians.

Participants were excluded based on the following criteria:

1. Individuals with MPS or OI who declined to undergo clinical dental examination or were uncooperative, including their parents or guardians.
2. Individuals with MPS or OI and their parents or guardians who refused to sign the informed consent form.

### 2.4. Data Collection

Data collection involved the completion of a questionnaire with closed-ended questions by the parents/guardians, and oral examinations of the children and adolescents. The questionnaire was developed based on the instruments employed by Prado et al. (2019) [8] and Teixeira et al. (2021) [11], addressing individual and sociodemographic characteristics (sex, age, skin color, and family income) as well as the medical and dental history of the children/adolescents (toothache in the previous 12 months and referral to a dentist by a health care provider). The oral examinations were performed by three previously trained and calibrated dentists.

Medical records were retrieved to confirm the specific type of condition (MPS or OI) for each participant. The referral to a dentist by the multiprofessional team was determined based on the answer to the following question: “Has any health professional ever recommended a dentist for your child?” Toothache was recorded when having occurred in the 12 months prior to the application of the questionnaire [8,16]. The ethno-racial characteristic was recorded using the criteria established by the Instituto Brasileiro de Geografia e Estatística-IBGE (Brazilian Institute of Geography and Statistics) for skin color/race: white, black, yellow, brown, or indigenous [17]. Family income was determined by the total monthly income of all economically active members of the family of each individual, using the monthly minimum wage as reference (USD 219.00 in January 2024).

The oral examination was performed with the participant positioned on a chair under artificial light (Petzl Zoom head lamp^®^, Petzl America, Clearfield, UT, USA). The three examiners used mouth mirrors (Duflex^®^ nº5), Community Periodontal Index probes (Golgran^®^, São Paulo, SP, Brazil), and personal protective equipment. No radiographs were taken.

Malocclusion was recorded based on the presence of at least one of the following conditions: overjet (increased/protrusion, anterior crossbite, absent), overbite (increased bite/deep, anterior open bite, absent, top) and posterior crossbite [8,9,17]. The following dental anomalies were investigated—conoid teeth, agenesia, microdontia, rotation, developmental defects of enamel (DDE), and dentinogenesis imperfecta. Dental agenesia was recorded as a possible diagnosis, since only a clinical examination was performed [8,15,18]. Caries experience was investigated following the WHO diagnostic criteria through the DMFT/dmft indices (sum of decayed, missing, and filled teeth in the permanent and primary dentitions, respectively) [13,14]. Gingivitis was investigated from an analysis of the contour and color of the gingival tissue [19].

### 2.5. Training and Calibration Exercises

Three examiners underwent training and calibration exercises. First, a theoretical training was performed and involved the analysis of images of the clinical conditions investigated. The inter- and intra-examiner diagnostic agreement was verified with a one-week interval between the two training periods. Cohen’s Kappa coefficients in this phase ranged from 0.74 to 1.00, demonstrating that the examiners were duly trained in the theoretical phase. The second phase consisted of practical training. Calibration was performed on two occasions with a 10-day interval between examinations. The calibration phase involved five children/adolescents with MPS or OI and five children and adolescents without rare genetic diseases under care at the hospital affiliated with the Federal University of Minas Gerais in the city of Belo Horizonte, Minas Gerais state, Brazil. Cohen’s Kappa coefficients for the clinical conditions investigated ranged from 0.78 to 1.00.

### 2.6. Pilot Study

After training and calibration, a pilot study was conducted involving five children and adolescents with one of the rare diseases investigated and their parents/guardians. The results of the pilot study revealed no need to alter the methods proposed for the study. Therefore, the participants in this step were included in the sample of the main study.

### 2.7. Statistical Analysis

The data were analyzed with the aid of the Statistical Package for the Social Sciences (SPSS for Windows, version 26.0, IBM Inc., Armonk, NY, USA). Unadjusted and adjusted logistic regression analyses were performed to identify the independent impact of each variable of interest. The dependent variable was referral to a dental service by the multiprofessional team. The independent variables were age, sex, skin color, family income, type of rare disease, toothache in the previous 12 months, malocclusion, gingivitis, caries experience and dental anomalies. Independent variables with a *p*-value < 0.25 in the bivariate analysis were incorporated into the multiple logistic model in decreasing order using the stepwise backward method. The goodness-of-fit of the model was assessed using the Hosmer–Lemeshow test. Influential values were identified based on Cook’s distance measure, and multicollinearity was investigated considering the variation inflation factor (VIF).

## 3. Results

The sample was composed of 87 children/adolescents with rare genetic conditions and their parents/guardians. The age of participants ranged from 2 to 21 years. The majority [59 (67.8%)] were between 2 and 12 years old, while 28 (32.2%) were aged 13 to 21 years. The mean age was 10.4 ± 5.6 years. A total of 17.3% of the children/adolescents had never been to a dentist. However, the majority (70.1%) had been referred to a dentist by the multiprofessional team at some time in life. An overview of the sample’s characteristics is outlined in Table 1.

According to the parents/guardians, the most recent dental appointment was within one year among 46.0% of the children and adolescents, one to two years prior to the study among 21.8% and more than two years prior to the study among 14.9%. The reason for most dental appointments was oral prophylaxis or preventive maintenance (62.1%), followed by restorative/periodontal/surgical treatment (10.3%), other reasons (6.9%), and toothache (3.4%).

With regard to counseling from the multiprofessional team to take the child/adolescent to a dentist, based on the reports of the parents/guardians, 33.3% received such counseling from a physician, 36.8% from other health care providers, and 29.9% never received counseling from any health care provider to take their children to a dentist.

Table 2 displays the results of the bivariate and multivariate analyses of factors associated with referrals by the multiprofessional team to a dentist for children/adolescents with rare genetic diseases. Sex (*p* = 0.151), toothache in the previous 12 months (*p* = 0.019), malocclusion (*p* = 0.080), and dental caries experience (*p* = 0.023) were incorporated into the multivariate logistic regression model. Only the variables sex and toothache in the previous 12 months remained associated with referral to dental services by the multiprofessional team. Female participants were 2.67 more likely to be advised to go to a dentist by the multiprofessional team (95% CI: 0.96–7.42). Children/adolescents with a history of toothache in the previous 12 months were 7.74 times more likely to be advised to seek dental care than those without a history of toothache in the previous 12 months (95% CI: 1.61–37.14).

The residual analysis indicated Cook’s distance values less than 1. The analysis of multicollinearity revealed VIF values less than 10. The Hosmer–Lemeshow test demonstrated that the final model was considered adequate (*p* = 0.820).

## 4. Discussion

The present study identified some factors associated with referrals to dental services by a multidisciplinary team that assist individuals with MPS and OI. Among the analyzed variables, sex and occurrence of toothache remained significantly associated with dental referral. Specifically, female participants with both rare diseases and those who had experienced toothache in the past 12 months were more likely to be referred for dental care by the multiprofessional team. These findings highlight how both sociodemographic and clinical aspects influence the decision-making process of health care professionals in referring patients to dental services.

As girls with rare diseases were more likely to be referred to dental services by other health care providers, several justifications may explain this finding. Before discussing this result, it is necessary to point out that the two rare diseases investigated in the present study (MPS and OI) are characterized by a high frequency of malocclusion and dental abnormalities [8]. The sample in this study was mainly composed of individuals with OI, who do not have any intellectual deficiency, unlike what occurs in MPS and a recent and pioneering epidemiological survey showed that in Brazil, just over half of people with rare diseases are female [6].

Besides the presence of malocclusion, OI is associated with a high frequency of dentinogenesis imperfecta [11] and these two characteristics directly compromise the esthetics of the teeth. Due to being more concerned with physical appearance, girls tend to place more value on smile esthetics, especially when becoming adolescents. Beginning with adolescence, girls tend to have clearer, healthier beliefs with regard to oral health in comparison to boys. Thus, health care providers are probably more attentive to this esthetic issue of girls, increasing the likelihood of a referral to the dentist.

Researchers have reported that some factors may explain the greater likelihood of health care providers to counsel female patients to seek dental care in comparison to male patients. It is possible that the main factor is associated with the cultural issue of the majority of women being more interested in health, oral hygiene, appearance, and beauty than men. Men tend to seek medical and dental services less, visiting a dentist mainly due to an acute problem and not necessarily for the prevention of oral diseases [20,21].

Another factor that may explain the greater referral of girls to dental services is associated with the chance that female patients are more vulnerable to dental caries. Studies have shown a possible association between sex and caries, indicating the possibility that girls have a greater susceptibility to caries due to the earlier tooth eruption pattern in comparison to boys. Lastly, the manifestation of caries is dependent on the length of exposure to etiological factors [21]. The greater likelihood of carious lesions in the female sex may also be explained by salivary composition and flow as well as genetic variations (influence on the structure and proteins of tooth enamel) and cultural factors [21].

The findings also indicate that a history of toothache within the last 12 months increased the likelihood of children/adolescents with rare genetic disorders being referred to dental services by the multiprofessional team in comparison to those without a history of toothache. This demonstrates that the parents of children and adolescents with rare diseases often forget preventive oral health care due to the various other needs of their children and that dental care is only sought when invasive curative measures are required. The literature reports that, although oral problems can cause pain, infections, respiratory complications, chewing problems and speech problems, oral health care is seen as being of lower priority in comparison to other forms of health care for individuals with rare genetic diseases [22,23].

Acute pain generates stress during dental treatment related to both the diminished cooperation of the patient and the complexity of the procedure, which is generally more invasive [22,24]. This situation can be avoided by early, preventive care and health promotion. Poor oral health status and episodes of toothache can exert a negative impact on quality of life.

The high prevalence of oral problems in individuals with rare diseases underscores the importance of the team that provides care to be attentive to the oral health status of their patients. When one of these health care providers advises parents to take their child/adolescent to the dentists, parents tend to follow this advice, with greater oral health gains achieved when this occurs earlier. For early care to occur with greater frequency, it is fundamental for the multiprofessional team that provides care for these patients to be aware of the role of dentistry in the achievement of better quality of life for this population.

Some weaknesses and strengths of the present study should be considered. A cross-sectional design was chosen, and it limits the ability to establish causal relationships between the presence of rare genetic diseases and the outcomes observed. A convenience sampling strategy was adopted, and no prior sample size calculation was conducted. This limitation is primarily due to the low prevalence of MPS and OI, which poses significant challenges to recruiting large samples. Moreover, the use of a questionnaire is accompanied by the risk of recall bias. However, some strong points should also be considered. The study shows the importance of better integration among the health professionals who provide care for patients with rare diseases. Continued, permanent educational measures, programs, and health policies can be suggested based on this study. Relationships among the professionals involved in the promotion of health for patients with rare diseases should be strengthened to achieve positive results in the lives of these individuals and their families.

The present study addresses the role of the dentist within the multidisciplinary team caring for children/adolescents with rare conditions, emphasizing the importance of individualized oral health strategies. By considering each patient’s genetic condition and specific clinical needs, the study highlights how dentistry contributes to the principles of personalized medicine. The study reinforces the integration of dental care into personalized, patient-centered approaches that aim to improve quality of life and long-term outcomes. Although public health policies in Brazil for individuals with rare genetic diseases continually advance, difficulties remain in the interaction of dentistry with other fields that comprise health teams. Understanding what makes the multiprofessional team refer children and adolescents with rare genetic diseases to oral health services is essential to the establishment of actions directed at this objective.

## 5. Conclusions

The findings of the present study showed that referrals to dental treatment by the multidisciplinary team that assist children and adolescents with MPS and OI were significantly associated with female sex and history of toothache in the past 12 months. The results highlight that clinical symptoms such as dental pain remain the main trigger for dental referrals, suggesting a curative rather than preventive approach. The inclusion of dentists in the multiprofessional team is essential to ensure early identification of oral health needs and promote preventive care and integrated management of individuals with rare genetic diseases.

## Figures and Tables

**Table 1:** Sociodemographic and clinical oral characteristics of children/adolescents with MPS and OI (*n* = 87).

Variables	Absolute Frequency (N)	Relative Frequency (%)
Age (years)		
2–12	59	67.8
13–21	28	32.2
Sex		
Male	50	57.5
Female	37	42.5
Skin color		
White	30	34.5
Non-white (black/brown/yellow)	57	65.5
Family income ^a^		
≤monthly minimum wage	14	16.1
>monthly minimum wage	73	83.9
Rare disease		
MPS	26	29.9
OI	61	70.1
Past dental experience		
Yes	72	82.7
No	15	17.3
Toothache in the previous 12 months		
No	64	73.6
Yes	23	26.4
Referral to a dentist by a professional		
Yes	61	70.1
No	26	29.9
Malocclusion		
Present	73	83.9
Absent	14	16.1
Gingivitis		
Present	26	29.9
Absent	61	70.1
Dental caries experience		
Present (DMFT/dmft > 1)	46	52.9
Absent (DMFT/dmft = Zero)	41	47.1
Dental anomalies		
Present	75	86.2
Absent	12	13.8

^a^ Monthly minimum wage in Brazil = USD 219.00 in January 2024.

**Table 2:** Factors associated with referral to dental services by the multiprofessional team for children/adolescents with rare genetic diseases (*n* = 87).

Independent Variables	Counseling by Health Professional to Visit Dentist (%)	OR (95% CI) Unadjusted	*p*-Value	OR (95% CI) Adjusted	*p*-Value
Age (years)					
2–12	67.8	1	0.494		
13–21	75.0	1.43 (0.52–3.93)			
Sex					
Male	64.0	1	0.151	1	0.061
Female	78.4	2.04 (0.77–5.39)		2.67 (0.96–7.42)	
Skin color					
White	70.0	1	0.986		
Non-white	70.2	1.01 (0.38–2.65)			
Family income ^a^					
≤monthly minimum wage	64.3	1	0.604		
>monthly minimum wage	71.2	1.37 (0.41–4.59)			
Rare disease					
MPS	29.9	1	0.906		
OI	70.1	1.06 (0.39–2.88)			
Toothache < 12 months					
No	62.5	1	0.019	1	0.011
Yes	91.3	6.30 (1.36–29.27)		7.74 (1.61–37.14)	
Malocclusion					
Absent	16.1	1	0.080		
Present	83.9	2.82 (0.89–9.17)			
Gingivitis					
Present	29.9	1	0.906		
Absent	70.1	1.06 (0.39–2.88)			
Caries experience					
Present (DMFT/dmft > 1)	62.5	1	0.023		
Absent (DMFT/dmft = 0)	83.9	2.91 (1.12–7.59)			
Dental Anomaly					
Absent	66.7	1	0.779		
Present	70.7	1.21 (0.33–4.42)			

OR: odds ratio/CI: confidence interval/^a^ Monthly minimum wage in Brazil = USD 219.00 in January 2024.

## Data Availability

The original contributions presented in this study are included in the article. Further inquiries can be directed to the corresponding author.

## Abbreviations

The following abbreviations are used in this manuscript:

MPS	Mucopolysaccharidosis
OI	Osteogenesis Imperfecta
OR	Odds ratio
CI	Confidence interval
